# A New Mouse Model for Marfan Syndrome Presents Phenotypic Variability Associated with the Genetic Background and Overall Levels of *Fbn1* Expression

**DOI:** 10.1371/journal.pone.0014136

**Published:** 2010-11-30

**Authors:** Bruno L. Lima, Enrico J. C. Santos, Gustavo R. Fernandes, Christian Merkel, Marco R. B. Mello, Juliana P. A. Gomes, Marina Soukoyan, Alexandre Kerkis, Silvia M. G. Massironi, José A. Visintin, Lygia V. Pereira

**Affiliations:** 1 Laboratório de Genética Molecular do Departamento de Genética e Biologia Evolutiva, Universidade de São Paulo, São Paulo, Brazil; 2 Departamento de Imunologia do Instituto de Ciências Biomédicas, Universidade de São Paulo, São Paulo, Brazil; 3 Departamento de Reprodução Animal da Faculdade de Veterinária e Zootecnia, Universidade de São Paulo, São Paulo, Brazil; University Hospital Vall d'Hebron, Spain

## Abstract

Marfan syndrome is an autosomal dominant disease of connective tissue caused by mutations in the fibrillin-1 encoding gene *FBN1*. Patients present cardiovascular, ocular and skeletal manifestations, and although being fully penetrant, MFS is characterized by a wide clinical variability both within and between families. Here we describe a new mouse model of MFS that recapitulates the clinical heterogeneity of the syndrome in humans. Heterozygotes for the mutant *Fbn1* allele mgΔ^loxPneo^, carrying the same internal deletion of exons 19–24 as the mgΔ mouse model, present defective microfibrillar deposition, emphysema, deterioration of aortic wall and kyphosis. However, the onset of a clinical phenotypes is earlier in the 129/Sv than in C57BL/6 background, indicating the existence of genetic modifiers of MFS between these two mouse strains. In addition, we characterized a wide clinical variability within the 129/Sv congenic heterozygotes, suggesting involvement of epigenetic factors in disease severity. Finally, we show a strong negative correlation between overall levels of *Fbn1* expression and the severity of the phenotypes, corroborating the suggested protective role of normal fibrillin-1 in MFS pathogenesis, and supporting the development of therapies based on increasing *Fbn1* expression.

## Introduction

Marfan Syndrome (MFS; OMIM#154700) is an autosomal dominant disorder with pleiotropic phenotype variations involving the skeletal, ocular and cardiovascular systems [1], [2], [3]. The disease incidence is 1 in 10,000 with 25% of cases being sporadic [4]. In 1991, mutations in the *FBN1* gene (OMIM 134797), which encodes the fibrillin-1 protein, were genetically linked to the MFS phenotype [5], [6], [7]. Although MFS is completely penetrant [8], it presents a wide clinical variability [9]. Genotype-phenotype correlations in MFS have been complicated by the large number of unique mutations reported, as well as by clinical heterogeneity among individuals with the same mutation; this extensive clinical variability, even within families, suggests the presence of modifier genes [10].

In 1997, a murine model for MFS - mgΔ - was created to mimic the dominant-negative effect of fibrillin-1 mutations seen in MFS patients [11]. Approximately 6 kb of the *Fbn1* gene encompassing exons 19–24 were replaced by a neomycin-resistance expression cassette (*neoR*), resulting in a protein monomer missing 272 residues. Surprisingly, heterozygote animals were histologically indistinguishable from wild-type mice, suggesting absence of the dominant-negative effects seen in MFS patients. In contrast, homozygote animals died before the second week of life, due to cardiovascular failure. Expression analysis showed that the mgΔ allele had a 90% lower transcript level compared to the normal *Fbn1* allele. Accordingly, it was postulated that the *neoR* cassette sequence had interfered with the mutant allele expression, consequently restricting the dominant-negative effect of the mutation.

Here we report the generation of a novel variant of the mgΔ mouse model in which the same mutant *Fbn1* allele is present, but with *neoR* flanked by lox-P sequences (mgΔ^loxPneo^), allowing Cre-recombinase-mediated deletion of the resistance cassette [12]. Unexpectedly, this construct now resulted in heterozygous mgΔ^loxPneo^ mice presenting some aspects of the MFS phenotype, including aortic, skeletal and respiratory system manifestations, before the removal of *neoR* sequence. Moreover, these phenotypes differ significantly between two different isogenic mouse strains, C57BL/6 (B6) and 129/Sv, and also vary within the 129/Sv background. Therefore, in addition to modeling the clinical manifestations of MFS disease, the mgΔ^loxPneo^ mouse model is an experimental system in which both the genetic background and epigenetic contributions to MFS clinical variability can be evaluated.

## Results

### Animal development

**Figure 1:**
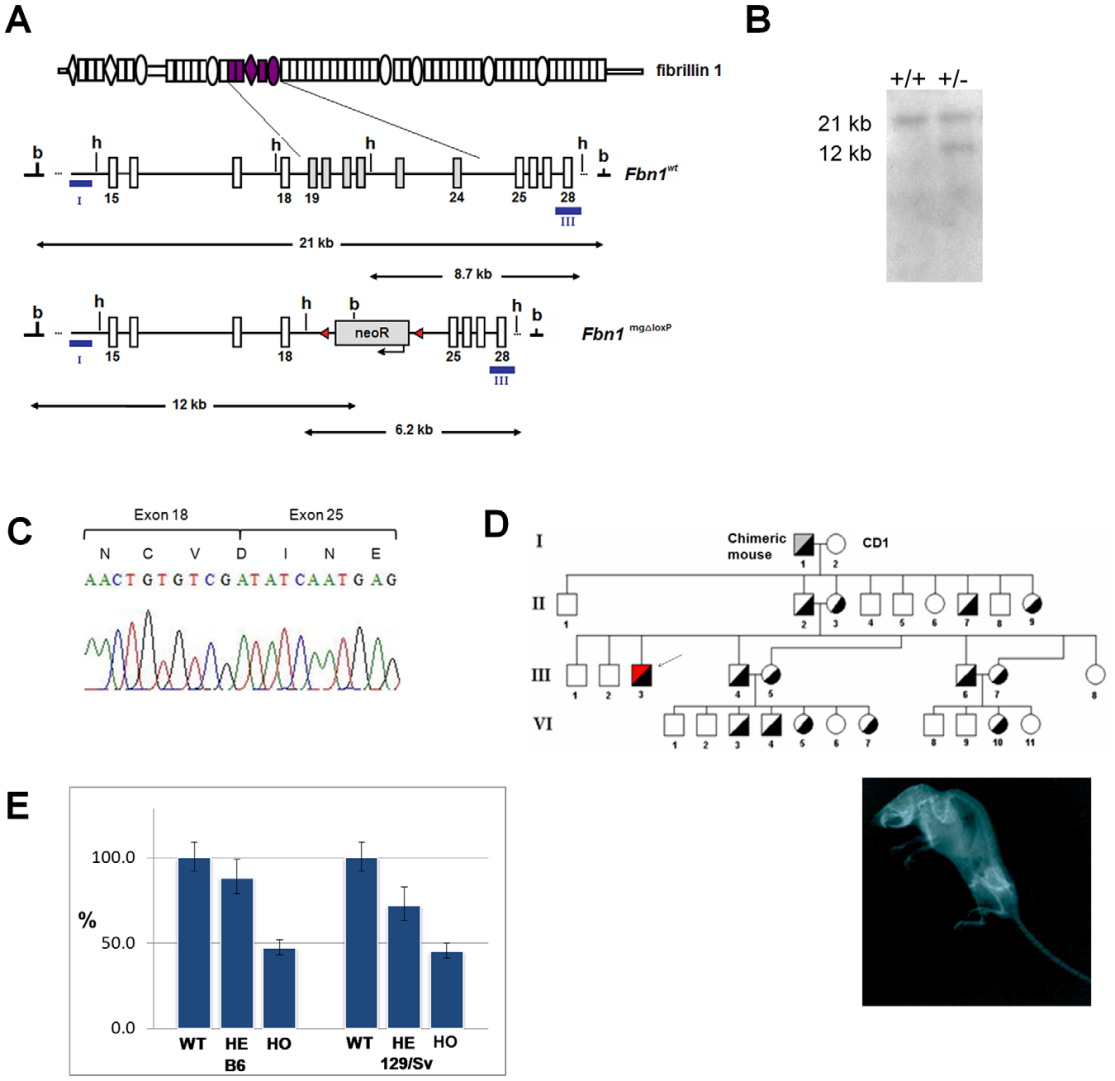
Targetting of *Fbn1*. (A) Scheme of *Fbn1* targeting. From top to bottom, fibrillin1 protein with the internally deleted region (residues 770–1042); schematic representation of the corresponding *Fbn1* gene region with the probes (I and III) used in the Southern-blot analysis, and the sizes of BamHI (b) and HindIII (h) fragments; and targeted *Fbn1* ^mgΔloxPneo^ allele with the neoR flanked by lox-P sequences (triangles). (B) Southern blot of genomic DNA from correctly targeted ES clone digested with BamHI, and hybridized to probe I. (C) sequence of the cDNA from the *Fbn1* ^mgΔloxPneo^ allele showing the junction of exons 18 and 25; (D) Heredogram of founder heterozygotes in a mixed 129/Sv/CD-1 background. Hetreozygotes were phenotypically normal until the F3, where one severely affected heterozygote was found (arrow), whose X-ray is shown. (E) Relative quantification of total *Fbn1* mRNA levels in wild-type (WT), heterozygous (HE) and homozygous (HO) fetal fibroblasts by Real Time RT-PCR analysis (P<0.05).

Cells from correctly targeted ES cell clones were aggregated to morulas, and used to produce chimeric male mice that, after mating with CD1 females, resulted in transmission of the mgΔ^loxPneo^ *Fbn1* allele to a proportion of the F1 generation (Figure 1A). RT-PCR and sequencing analysis of skin RNA from heterozygous animals confirmed the in-frame deletion of exons 19–24 in transcripts derived from the targeted allele (Figure 1B–C). As observed in the original mgΔ strain, the heterozygous mgΔ^loxPneo^ from the F1 did not present any apparent phenotype. However, in subsequent crosses between F1 heterozygotes, we obtained some heterozygous animals with a very severe phenotype, characterized by deformities of the spine at 2 months of age (Figure 1 D). From a total of 47 heterozygous mice, four presented the mentioned feature, and died by 3 months of age of unknown causes presenting hemothorax, suggestive of aortic rupture. We also obtained four homozygous animals that died between 4 and 8 days of age. All these animals came from mixed 129/Sv and CD-1 backgrounds.

These results suggested that the phenotype variability could be associated with the different genetic backgrounds of the animals. To test this hypothesis, the mgΔ^loxPneo^ allele was put into two different isogenic backgrounds, namely the 129/Sv and B6 strains. Haplotype analysis of a large panel of microsatellite markers confirmed the congenic status of mice after 14 generations (data not shown).

Real-time RT-PCR analysis of embryonic day 13 fibroblasts from both strains showed 47%±5 (P<0.05) of *Fbn1* mRNA levels in homozygous, and 78%±10 (P<0.05) in heterozygous fibroblasts when compared to wild-type cells (Figure 1E). Therefore, the level of expression of the mgΔ^loxPneo^ allele is significantly higher than the 10% level observed in the original mgΔ allele [11], which may explain the differences in phenotypes seen in heterozygotes between the two models.

**Figure 2:**
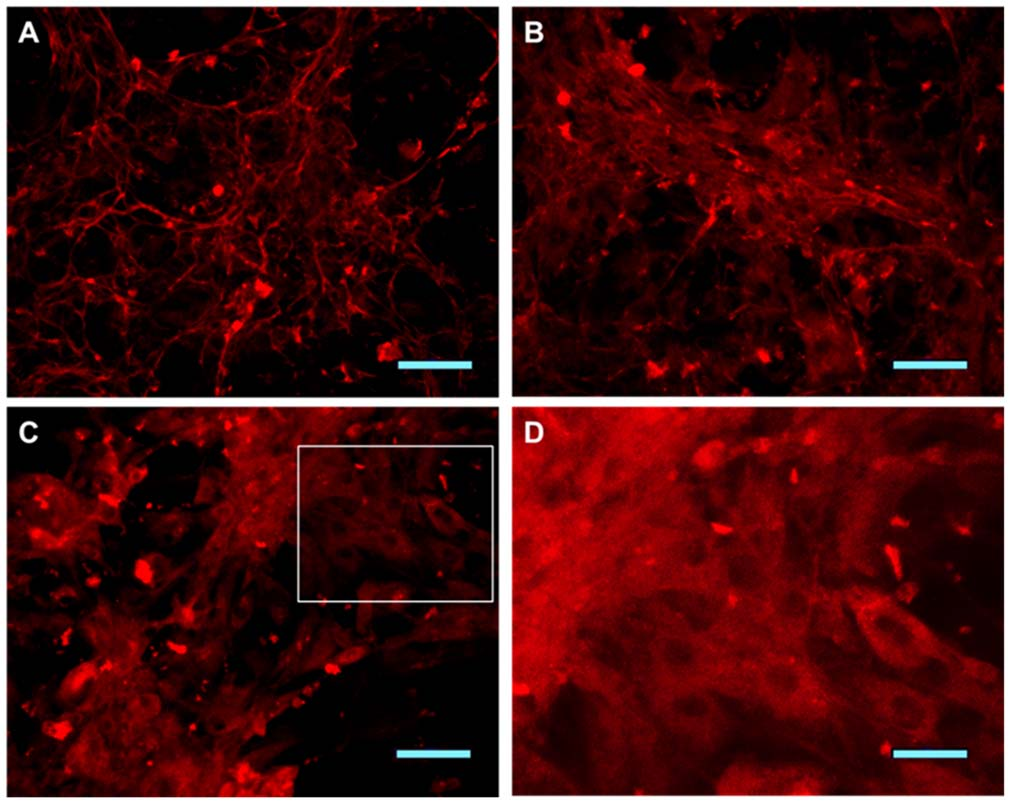
Immunofluorescence of cultured fetal fibroblasts. (A) Wild-type, (B) heterozygous and (C) homozygous fibroblasts immune-stained for fibrillin-1 (scale bars 50 µm). In contrast to the elaborate network of immunoreactive fibrillin-1 seen in control lines, heterozygous and homozygous cell cultures show a diffuse pattern of immunoreactive material. (D) Representative field at higher magnification to show intracellular deposition of mutant protein (scale bar 25 µm).

Immunohistochemical comparison of cultured fetal fibroblasts from B6 animals revealed qualitative differences in fibrils between wild-type and mutant cells (Figure 2). In contrast to the elaborate network of immunoreactive fibrillin-1 seen in control lines (Figure 2A), heterozygous cells present fibers spread over a diffuse background (Figure 2B), while homozygous cell cultures show a diffuse pattern of immunoreactive material (Figure 2C) similar to that reported in cultured fibroblasts from homozygous mgΔ, and heterozygous Tight Skin models [11], [13]. In the homozygous mutant fibroblasts we also observed an apparent intracellular deposition of mutant protein, evidenced by the visualization of cells with nuclear regions delimitated by marked protein (Figure 2D). The changes observed in the immunofluorescence assays indicate that a portion of the mutant molecules may be retained inside the cell, which in turn, could be a significant factor contributing to the pathogenesis of MFS [14].

### Phenotypes

**Figure 3:**
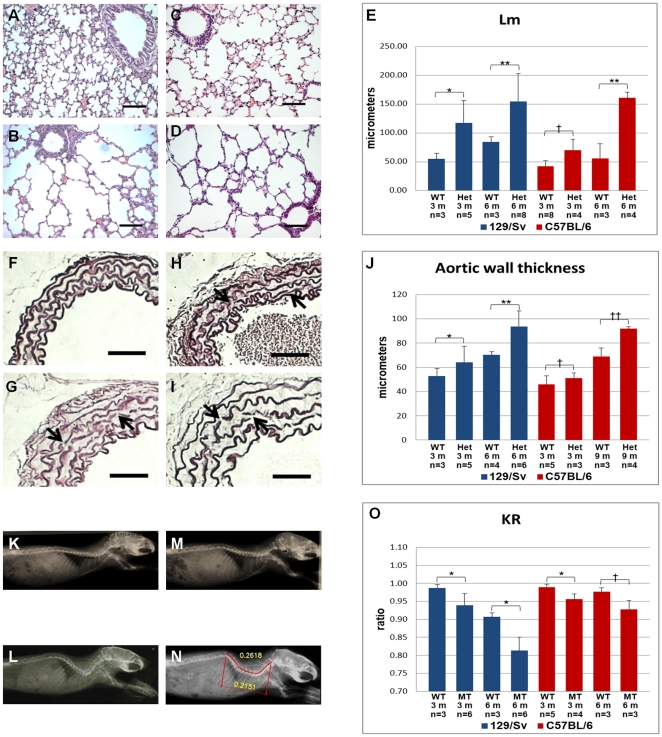
Phenotypic variability between strains. (A to D) Histologic analysis of lung (HE staining), scale bars 100 µm. (A) wild-type mice show normal airspace caliber; (B) 129/Sv heterozygous mouse by 3 months of age shows diffuse distal airspace widening; B6 heterozygous mice present (C) mild alterations by 3 months of age, and (D) severe phenotype by 6 months of age; (E) Average mean linear intercept is greater for mutant mice than for wild-type (*P<0.02, **P<0.04, †P<0.01); (F–I) Histologic analysis of thoracic aorta (Weigert coloration), scale bars 100 µm. (F) wild-type, (G) 129/Sv heterozygote at 3 months of age, B6 heterozygote at (H) 3 months and (I) 9 months of age. Note diffuse fragmentation of elastic fibers (arrows). (J) Average thickness of the thoracic aortic media (*P<0.05, **P<0.007, †P<0.18, ††P<0.03); (K–N) X-ray analysis. (K) Wild-type, (L) 3 months old 129/Sv heterozygote, B6 heterozygote at (M) 3 months and (N) 9 months of age. (O) Average KR, used to quantify skeletal phenotype (*P<0.01, †P<0.04). The number of animals analyzed in each group (n) and the respective age (3 m, 6 m, and 9 m indicate 3, 6 and 9 months, respectively) are shown.

Heterozygous mice from both strains have normal lifespan and reproductive capacity, but display some of the classic MFS phenotypes (Figure 3). Pulmonary alterations include enlargement of peripheral air space (respiratory bronchioles and alveoli), and destruction of alveolar wall structures, characterizing pulmonary emphysema (Figure 3A–D). We also detected a large amount of infiltrating mononuclear cells, indicating a chronic inflammation process in the lung. The cardiovascular phenotypes include thickening of the aortic media, disruption/degradation of the elastic fibers (Figure 3F–I), but no inflammatory cells were detected. Finally, mutant animals also presented skeletal manifestations, mostly kyphosis (Figure 3K–N).

In order to characterize the phenotypic variability in the two strains, we quantified the phenotypes in the three affected organ systems. Analysis of the mean linear intercept (Lm) revealed changes on the average size of alveoli in 129/Sv heterozygotes (Figure 3E). By 3 months of age, these animals present an Lm significantly higher (p<0.02) than wild-type mice, and this difference became more pronounced with age. In contrast, B6 mice at 3 months of age presented milder (although significant, p<0.01) pulmonary alterations, and only at 6 months of age did the alterations become severe.

The vascular phenotype was quantified by measuring the thickness of the aortic media in heterozygous animals. As observed in the lung, at 3 months of age 129/Sv heterozygotes exhibited a significantly enlarged media when compared to wild-type, and this difference increased with age (Figure 3J). In contrast, in the B6 background heterozygous mgΔ^loxPneo^ mice were asymptomatic at 3 months of age, whereas by 9 months they presented severe alterations indistinguishable from those of 129/Sv heterozygotes at the same age.

Quantification of the skeletal manifestations was performed by calculating the ratio between the linear distance and the length from the first cervical vertebrae to the last thoracic vertebrae (KR) (Figure 3N). As with the other phenotypes, heterozygous animals from the 129/Sv strain manifested a more severe skeletal phenotype earlier than those from the B6 background (Figure 3O). Together, these data show that the main differences observed in disease manifestation between the two strains is the age of onset of symptoms, which is delayed in B6 animals.

Finally, when present in homozygocity the mutation is lethal during gestation in both strains. Crossings between heterozygotes produced 30 (42%) wild-type animals and 41 (58%) heterozygous offsprings. Homozygous mutant embryos were identified only prior to embryonic day 13.

### 129/Sv phenotypic variability

**Figure 4:**
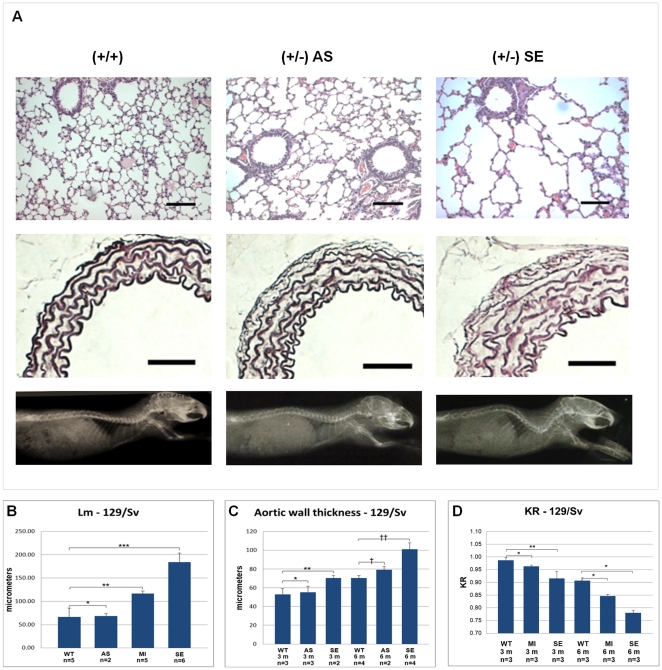
Phenotypic variability within the 129/Sv strain. (A) Comparison of histological slides and radiography of 129/Sv heterozygotes at 3 months of age show phenotypic variation in this strain. Wild-type (+/+), asymptomatic (AS) and severely affected (SE) heterozygous mice. (B) Average mean linear intercept at 3 and 6 months of age (*P<0.42, **P<0.0006, ***P<0.006). (C) Average thickness of thoracic aortic media at 3 and 6 months of age (*P<0.33, **P<0.04, †P<0.8, ††P<0.01). (D) Average KR at 3 and 6 months (*P<0.03, **P<0.04). (MI) Mildly affected animals. The number of animals analyzed in each group (n) and the ages (3 m and 6 m represent 3 months and 6 months, respectively) are shown.

Interestingly, while the different phenotypes are homogeneous among B6 heterozygotes (Figure 3E, J and O), animals from the 129/Sv strain present a wide clinical variability before 6 months of age, with phenotypes varying from mild to very severe (Figure 4A). Based on the average Lm in alveoli, we scored thirteen 129/Sv heterozygotes between 3 to 6 months of age in 3 clinical categories (Figure 4B): compared with wild-type animals (Lm = 66.75±18.26 µm), 15% were classified as asymptomatic (Lm = 68.45±5.2 µm; P<0,42); 38% as carrying moderate alterations (Lm = 116.44±5.46 µm; P<0.0006) and 46% as presenting severe alterations (Lm = 183.86±19.84 µm; P<0.006).

The aortic phenotype also exhibited variability in the 129/Sv heterozygotes at 3 months of age (Figure 4C), allowing the classification of phenotypes in 2 categories: 60% were defined as asymptomatic (55.21±6.46 µm versus 61.61±11.08 µm in wild types; P = 0.33), and 40% were classified as presenting moderate changes (77.75±2.59 µm; P<0.04). At 6 months of age, 33% of the heterozygotes were classified as asymptomatic (79.10±3.43 µm versus 70.34±2.72 µm in wild types; P = 0.80); and 66% were scored as severely affected (101.85±6.92 µm; P<0.01).

A similar variability was observed in the skeletal phenotype in the 129/Sv strain, where heterozygotes with 3 months of age displayed different degrees of kyphosis, allowing the classification of heterozygotes into distinct classes of mild and severe, according to the animal age (Figure 4D).

When analyzed together, the phenotypic variations in the 129/Sv background show a strong correlation (|R|≥0.70), i.e., animals with more severe manifestations in the pulmonary tissue also have a more thickened aortic wall, as well as a more severe kyphosis (Figure 4A).

### Molecular analysis

**Figure 5:**
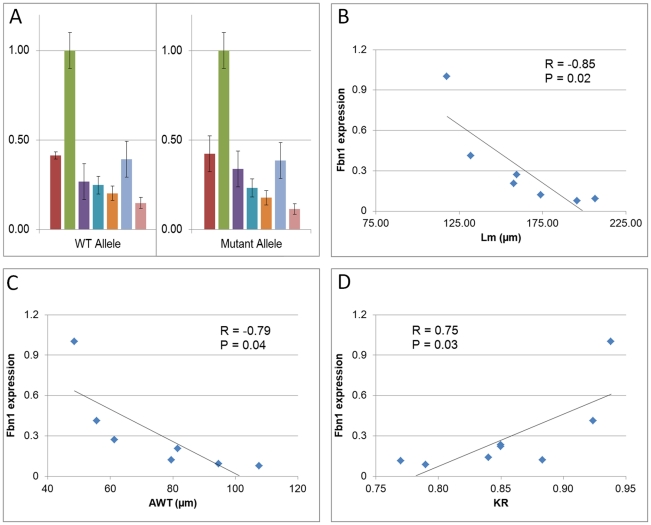
Correlation between *Fbn1* expression and phenotype severity in 129/Sv heterozygotes. (A) Real-time RT-PCR analysis of wild-type (WT – left panel) and mutant (right panel) *Fbn1* alleles in heterozygous 129/Sv animals. Individual animals are represented by different colored bars. For each animal three technical replicas were performed. Note that, despite the expression variability among different animals, the ratio between mutant and normal *Fbn1* transcripts is constant. (B to D) Correlation between level of expression of total *Fbn1* (Y axis) and the severity of (B) lung, (C) vascular (AWT: aortic wall thickness), and (D) skeletal phenotypes in heterozygous 129/Sv animals. Diamonds correspond to the animals represented in (A). Pearson's correlation coefficients (R) and the statistical significance (P) are shown. Note strong correlation (|R|≥0.75) between levels of total *Fbn1* expression and severity of the phenotype in the three affected systems.

The levels of normal and mutant fibrillin-1 transcripts have been associated with the severity of the disease in humans and mice [15], [16]. Therefore, we analyzed the expression of the normal and mgΔ^loxPneo^-mutant *Fbn1* alleles in differently affected animals of the 129/Sv strain to evaluate if there was any correlation with the corresponding phenotypes. Real-time RT-PCR from lung RNA showed a wide variability in the levels of expression of normal and mutant *Fbn1* alleles in 129/Sv heterozygotes (Figure 5A). Furthermore, the analysis of heterozygotes in the 129/Sv background revealed that the ratio between mutant and normal *Fbn1* transcripts is similar among animals with different phenotypes (Figure 5A). However, there is a strong negative correlation (|R|≥0.75) between the overall *Fbn1* expression and the severity of the phenotypes, i.e., mice with lower expression of *Fbn1* gene tend to have more severe phenotypes in the three affected systems (Figure 5B–D).

## Discussion

The first knockout mice to model MSF were homozygous for hypomorphic alleles of *Fbn1* gene: the mgΔ and mgR models [11]. While the mgΔ expresses a truncated form of fibrillin-1 at very low levels resulting in early post-natal lethality, mgR homozygotes produce low levels of normal fibrillin-1, and recapitulate the adult lethal form of MFS [17]. A third model, the C1039G, is characterized by substitution of a cysteine by a glycine at residue 1039, in an EGF domain of fibrillin-1, one of the most common type of mutations in humans [16], [18]. Heterozygotes show deficiency in the deposition of microfibrils in the extracellular matrix, skeletal disorders, and progressive deterioration of the architecture of the aortic wall. The three mouse models have been characterized exclusively in the B6 inbred strain [11], [14], [24].

The mgΔ^loxPneo^ is a new mouse model for MFS that, in addition to recapitulating some important phenotypes of the human disease, presents the clinical variability characteristic of the syndrome. The main difference between this new mutation and the original mgΔ is the presence in the former of two loxP sites flanking *neoR* – both mutant alleles result in *Fbn1* transcripts with an in frame deletion of exons 19 through 24. Nevertheless, the mgΔ^loxPneo^ allele has a 47% increase in the level of expression when compared to the mgΔ mutant allele which, according to the dominant-negative model of pathogenesis for MFS, can explain the manifestation of the disease in mgΔ^loxPneo^ heterozygotes. We are currently breeding these animals with CRE-transgenic mice in order to eliminate *neoR* and possibly increase the level of mutant transcripts to that of the normal mRNA in the heterozygotes.

The mgΔ^loxPneo^ heterozygotes have phenotypes similar to the C1039G model [16], including conserved number of elastic lamellae in the aortic media despite overall enlargement of the aorta. However, this new model presents a chronic inflammation process in the lung, characterized by the presence of large number of mononuclear cell in the lung parenchyma, as observed in the hypomorphic homozygous mgR model [19].

We demonstrated the effect of the genetic background on the severity of the MFS phenotype by backcrossing the mgΔ^loxPneo^ allele into two different inbred mouse strains, and showing the earlier onset of the disease in heterozygotes in the 129/Sv background when compared to the B6 strain. This inter-strain phenotypic variability could be attributed to differences in the extent of interference of the *neoR* cassete in the expression of the mutant allele between the two congenic strains. However, we show that the level of expression of the mutant allele is 47% of the wild type *Fbn1* in both strains. Therefore, we believe that genetic factors modulating the difference in phenotype expression between the two strains can be relevant for the pathogenesis of MFS. Interestingly, the most severe phenotypes, including death at 3 months of age, were observed in the mixed 129/Sv-CD-1 animals, indicating the existence of important modifying alleles in the outbred CD-1 strain. The influence of the genetic background on the phenotypes has been reported in knockout mouse models of several human diseases, including cystic fibrosis and Huntington disease [20], [21], [22]. Furthermore, in some cases those animals have been successfully used for the identification of the respective genetic modifier [23], [24], [25]. The variability documented here in the mgΔ^loxPneo^ model for MFS may allow the identification of genetic modifiers underlying the clinical heterogeneity of the disease in humans.

In addition, we observed an unexpected wide clinical variability within the 129/Sv congenics, where severity of phenotypes varied greatly among heterozygotes. Moreover, we showed that there was a strong negative correlation between the levels of total *Fbn1* expression and the severity of the phenotype, while the ratio of mutant versus normal *Fbn1* expression did not vary among the differently affected animals. The protective effect of higher levels of normal fibrillin-1 in MFS has been proposed by Hutchinson et al. (2003), who showed a correlation between higher levels of the normal *FBN1* transcript and milder phenotypes in 3 affected individuals of the same family [10]. However, the *FBN1* mutation in that family lead to nonsense mediated decay of the mutant transcript, not being able to exert a dominant-negative effect. In contrast, heterozygotes in the congenic 129/Sv strain present the alleged protective role of higher levels of normal fibrillin-1 even in the presence of proportionally higher levels of mutant fibrillin-1. Therefore, although the presence of mutant monomers may increase the tissue damage found in individuals with MFS [26], the levels of normal fibrillin-1 seem to have the potential to modulate the pathogenic effects of mutated proteins. This in turn reinforces the hypothesis that boosting *FBN1* expression may be an efficient therapeutic strategy for MFS.

Finally, since the 129/Sv congenic were backcrossed more than 14 times, the difference in phenotypes observed among heterozygotes cannot be attributed to genetic heterogeneity. Phenotypic variability within mouse inbred strains has been reported for a number of conditions, and, in some cases has been associated with epigenetic modifications of specific loci [27]. Therefore, we propose that epigenetic factors contribute to the clinical variability of the mgΔ^loxPneo^ model in the 129/Sv background, and thus possibly to the MFS phenotype in humans. This hypothesis is currently being explored by analyzing DNA methylation in the differently affected 129/Sv animals.

In conclusion, mouse models for MFS have been fundamental in the discovery of unexpected disease mechanisms, and in the development of novel therapeutic interventions [28]. The new mouse model described here may allow the identification of genetic and epigenetic modifiers of the MFS phenotype, which in turn will lead to a better understanding of the clinical variability of the disease, and of the physiology of pulmonary, cardiovascular and skeletal systems.

## Materials and Methods

### Animals

All animals used were housed under controlled temperature and light conditions in pathogen-free environment at the Immunology Department of ICB USP experimentation housing facility. We analyzed 45 mutant animals and 20 wild-type animals, at three different ages and from two different mice strains. The C57BL/6 (B6) was chosen because it is the most widely used inbred strain, and all other extant mouse models for MFS are in that background. The 129/Sv was chosen because the ES cells used for gene targeting were derived from this strain. All animal experiments followed the protocols approved by the Institutional Animal Care and Use Committee of the Instituto de Biociências at USP. Protocol ID: CEA/IBUSP 020/2004.

### Development of the *mgΔ^loxPneo^* mouse model

The murine ES cell line USP-1 was used for gene targeting experiments [29]. Generation of positively targeted ES cell clones and production of chimaeric mice were performed as previously described [11]. The *Fbn1* ^mgΔloxPneo^ targeting vector was a modification of the previously described targeting vector used to create the mgΔ and mgR models [11], [17]. Two complementary 38-bp oligonucleotides with the loxP consensus sequence [12] were synthesized, annealed, and cloned flanking the *neoR* expression cassette, which was then used to replace the original *neoR* cassette of the mgΔ vector. Correctly targeted ES clones were identified by southern blot as described [11].

### Genotyping

DNA was extracted from a 0.5 cm piece of tail using Proteinase K (Promega) as described [30]. Each sample was submitted to two PCRs to identify the presence of the *Fbn1^mgΔloxPneo^* allele and the normal allele, which served as an internal reaction control. *Fbn1^mgΔloxPneo^* allele primers: forward 5′ -GAG GCT ATT CGG CTA TGA CT – 3′, reverse 5′ – CTC TTC GTC CAG ATC ATC CT – 3′. Cycling conditions were 94°C for 2.5 min, then 94°C, 57°C, 72°C for 1 min for 30 cycles in a 10 µl volume. *Fbn1^wt^* allele primers: forward 5′ – AAA CCA TCA AGG GCA CTT GC – 3′, reverse 5′ – CAC ATT GCG TGC CTT TAA TTC – 3′. Cycling conditions were 94°C for 2.5 min primary denaturation, then 94°C, 55°C, 72°C for 1 min for 30 cycles in a 10 µl volume.

### Immunofluorescence

Mouse embryonic fibroblasts (MEFs) were prepared from embryos at 13–14 days of gestation as described [31]. Cells were fixed in 4% paraformaldehyde in PBS for 20 min at 4°C and permeabilized in 0.05% Triton X-100 in PBS for 5 min. Non-specific binding was blocked with 10% FBS in PBS for 1 h at room temperature. Cells were incubated with pAb9543 (1∶1000 dilution) primary antibody [32] overnight at 4°C and with secondary antibody coupled to Cy3 for 1 h at room temperature. The fluorescence signals were examined using an Axiovert 200 (Carl Zeiss) and an ApoTome imaging system (Carl Zeiss).

### Histological analysis

Animals were sacrificed by cervical dislocation. Mouse tissues were processed as previously described [33]. Five-micron sections were stained with hematoxylin and eosin, and adjacent sections were assayed for Weigert coloration, specific for elastic fiber visualization. Slides were examined and photographed using an Axiovert 200 (Carl Zeiss).

### Phenotype quantification

Skeletal: A full body x-ray of each mouse was digitalized and the follow measurements were taken using AutoCAD software 2002: the cervical-thoracic segment length and the straight line distance of the same segment. With those measures we were able to establish a kyphosis ratio (straight distance/segment length), and use the ratio to score the animals according to the severity of the skeletal manifestation (the smaller ratio, the more severe the manifestation).

Aortic wall: The histological samples were photographed at 50× and 100× magnification, and the length of the inner and outer perimeters of the aorta were measured using the imageJ software [34]. From this we could estimate the inner and outer radius, and wall thickness of the aorta.

Lung: The size of alveolar airways was determined by measuring the linear intercept on H&E-stained lungs as previously described [35].

### Real-time RT-PCR

Total RNA was extracted from mouse lung and from mouse embryonic day 13 fibroblasts using TRIzol (Invitrogen Corp.). The RNA was treated with DNase according to the manufacture's instructions (Invitrogen Corp.). A total of 1 µg of total RNA was reverse-transcribed with SuperScript™ III First-Strand Synthesis System (Invitrogen Corp.). Wild type, mutant and total *Fbn1* mRNA levels were determined using real-time RT-PCR sequence detection (7500 Real Time System; Applied Biosystems). mRNA levels were normalized to *Actb* mRNA, and fold expression determined as previously described [36]. The following primers and probes sequences were designed using the PrimerExpress software (Applied Biosystems): WT forward 5′ – ACA TAA CTG GGA AAA ACT GTG TCG ATA – 3′, WT reverse 5′ – TTC CAG GTG TGT TTC GAC ATT GT – 3′, WT probe 5′ –TGT GCT GAA CAG TCT ACT– 3′; KO forward 5′ –GGG ATA TGA AGT AGA CAT AAC TGG GAA A– 3′, KO reverse 5′ – GAG GCT GGG TAT CAT CTT GCA – 3′, KO probe 5′ – ACT GTG TCG ATA TCA ATG– 3′; *Fbn1*Total forward 5′ –CCT GTG CTA TGA TGG GTT CA – 3′, *Fbn1*Total reverse 5′ – AGG TCC CAC TAA GGC AGA TGT – 3′; ACTB forward 5′ –ACGGCCAGGTCATCACTATTG – 3′, ACTB reverse 5′ –CAAGAAGGAAGGCTGGAAAAGA– 3′. Three technical replicates of each reaction were performed.

### Statistical Analyses

Pearson's Correlation Coefficient (R) was used to determine the correlation between the *Fbn1* gene expression and the severity of the phenotype, reflecting the degree to which the variables are related; weak correlation 0≤|R|≤0.29; moderate correlation 0.30≤|R|≤0.69; strong correlation |R|≥0.70. P<0.05 was deemed to be significant.

A nonparametric test, Mann Whitney test, was used to determine statistical significance for all tests other than the Pearson Correlation Coefficient. All statistical analyses were performed using MINITAB (R14). P<0.05 was deemed to be significant.

## References

[1] Pyeritz RE (1990). Marfan syndrome.. N Engl J Med.

[2] Silverman DI, Gray J, Roman MJ, Bridges A, Burton K (1995). Family history of severe cardiovascular disease in Marfan syndrome is associated with increased aortic diameter and decreased survival.. J Am Coll Cardiol.

[3] Pyeritz RE, McKusick VA (1981). Basic defects in Marfan syndrome.. N Engl J Med.

[4] Gray JR, Bridges AB, Faed MJ, Pringle T, Baines P (1994). Ascertainment and severity of Marfan syndrome in a Scottish population.. J Med Genet.

[5] Kainulainen K, Peltonen L (1991). Marfan gene discovered.. Ann Med.

[6] Kainulainen K, Steinmann B, Collins F, Dietz HC, Francomano CA (1991). Marfan syndrome: no evidence for heterogeneity in different populations, and more precise mapping of the gene.. Am J Hum Genet.

[7] Dietz HC, Cutting GR, Pyeritz RE, Maslen CL, Sakai LY (1991). Marfan syndrome caused by a recurrent de novo missense mutation in the fibrillin gene.. Nature.

[8] Pyeritz RE (1986). The Marfan syndrome.. Am Fam Physician.

[9] Pyeritz RE, Murphy EA, McKusick VA (1979). Clinical variability in the Marfan syndrome(s).. Birth Defects Orig Artic Ser.

[10] Hutchinson S, Furger A, Halliday D, Judge DP, Jefferson A (2003). Allelic variation in normal human FBN1 expression in a family with Marfan syndrome: a potential modifier of phenotype?. Hum Mol Genet.

[11] Pereira L, Andrikopoulos K, Tian J, Lee SY, Keene DR (1997). Targetting of the gene encoding fibrillin-1 recapitulates the vascular aspect of Marfan syndrome.. Nat Genet.

[12] Sunaga S, Maki K, Komagata Y, Ikuta K, Miyazaki JI (1997). Efficient removal of loxP-flanked DNA sequences in a gene-targeted locus by transient expression of Cre recombinase in fertilized eggs.. Mol Reprod Dev.

[13] Gayraud B, Keene DR, Sakai LY, Ramirez F (2000). New insights into the assembly of extracellular microfibrils from the analysis of the fibrillin 1 mutation in the tight skin mouse.. J Cell Biol.

[14] Whiteman P, Handford PA (2003). Defective secretion of recombinant fragments of fibrillin-1: implications of protein misfolding for the pathogenesis of Marfan syndrome and related disorders.. Hum Mol Genet.

[15] Buoni S, Zannolli R, Macucci F, Ansaldi S, Grasso M (2004). The FBN1 (R2726W) mutation is not fully penetrant.. Ann Hum Genet.

[16] Judge DP, Biery NJ, Keene DR, Geubtner J, Myers L (2004). Evidence for a critical contribution of haploinsufficiency in the complex pathogenesis of Marfan syndrome.. J Clin Invest.

[17] Pereira L, Lee SY, Gayraud B, Andrikopoulos K, Shapiro SD (1999). Pathogenetic sequence for aneurysm revealed in mice underexpressing fibrillin-1.. Proc Natl Acad Sci U S A.

[18] Ng CM, Cheng A, Myers LA, Martinez-Murillo F, Jie C (2004). TGF-beta-dependent pathogenesis of mitral valve prolapse in a mouse model of Marfan syndrome.. J Clin Invest.

[19] Neptune ER, Frischmeyer PA, Arking DE, Myers L, Bunton TE (2003). Dysregulation of TGF-beta activation contributes to pathogenesis in Marfan syndrome.. Nat Genet.

[20] Sweet A, Erickson RP, Huntington C, Dawson D (1992). A potential animal model for studying CF heterozygote advantage: genetic variation in theophylline-inducible colonic chloride currents among inbred strains of mice.. Biochem Med Metab Biol.

[21] Otsuru S, Hofmann TJ, Rasini V, Veronesi E, Dominici M (2010). Osteopoietic engraftment after bone marrow transplantation: Effect of inbred strain of mice.. Exp Hematol.

[22] Yang T, Huang YG, Ye W, Hansen P, Schnermann JB (2005). Influence of genetic background and gender on hypertension and renal failure in COX-2-deficient mice.. Am J Physiol Renal Physiol.

[23] Wheeler FC, Fernandez L, Carlson KM, Wolf MJ, Rockman HA (2005). QTL mapping in a mouse model of cardiomyopathy reveals an ancestral modifier allele affecting heart function and survival.. Mamm Genome.

[24] Dietrich WF, Lander ES, Smith JS, Moser AR, Gould KA (1993). Genetic identification of Mom-1, a major modifier locus affecting Min-induced intestinal neoplasia in the mouse.. Cell.

[25] Pu WT (2009). Identification of a cardiac disease modifier gene using forward genetics in the mouse.. PLoS Genet.

[26] Booms P, Tiecke F, Rosenberg T, Hagemeier C, Robinson PN (2000). Differential effect of FBN1 mutations on in vitro proteolysis of recombinant fibrillin-1 fragments.. Hum Genet.

[27] Rakyan VK, Blewitt ME, Druker R, Preis JI, Whitelaw E (2002). Metastable epialleles in mammals.. Trends Genet.

[28] Judge DP, Dietz HC (2008). Therapy of Marfan syndrome.. Annu Rev Med.

[29] Sukoyan MA, Kerkis AY, Mello MR, Kerkis IE, Visintin JA (2002). Establishment of new murine embryonic stem cell lines for the generation of mouse models of human genetic diseases.. Braz J Med Biol Res.

[30] Zangala T (2007). Isolation of genomic DNA from mouse tails.. J Vis Exp.

[31] Hogan A, Beddington R, Constantine F, Lacy E (1994). Manipulating the mouse Embryo: a laboratory manual..

[32] Reinhardt DP, Keene DR, Corson GM, Poschl E, Bachinger HP (1996). Fibrillin-1: organization in microfibrils and structural properties.. J Mol Biol.

[33] Andrikopoulos K, Liu X, Keene DR, Jaenisch R, Ramirez F (1995). Targeted mutation in the col5a2 gene reveals a regulatory role for type V collagen during matrix assembly.. Nat Genet.

[34] Muraishi H [Fundamental of medical image processing with personal computer system—development of Plugins by ImageJ].. Nippon Hoshasen Gijutsu Gakkai Zasshi.

[35] Dunnill MS (1962). Quantitative Methods in the Study of Pulmonary Pathology.. Thorax.

[36] Livak KJ, Schmittgen TD (2001). Analysis of relative gene expression data using real-time quantitative PCR and the 2(-Delta Delta C(T)) Method.. Methods.

